# Anatomical characterisation of an additional atrioventricular node artery contributing to the arterial complex of proximal conduction components: Bonapace’s septal branch or Kugel’s collateral artery

**DOI:** 10.1136/openhrt-2025-003520

**Published:** 2025-09-11

**Authors:** Tomokazu Kawashima

**Affiliations:** 1 Department of Anatomy, Toho University, Ota-ku, Tokyo, Japan

**Keywords:** Arrhythmias, Cardiac, Ablation Techniques, Translational Medical Research

## Abstract

**Background:**

Transient or permanent iatrogenic conduction disturbances from injury to arteries supplying the cardiac conduction system (CCS) have increased. A more comprehensive understanding of CCS arterial anatomy is essential for advancing electrophysiological studies. Compared with the sinus node (SN) artery, atrioventricular node (AVN) artery is more variable and difficult to identify because of the thicker surrounding myocardium. Arterial sources other than the main AVN artery branching near the cardiac crux—such as the Bonapace’s septal branch or Kugel’s collateral artery—have often been overlooked or regarded as atypical and remain poorly characterised.

**Methods:**

An alternative origin and course of the AVN artery were examined in 116 hearts via microdissection and in 14 additional hearts using serial histological sections of physiologically aged hearts without coronary artery occlusion.

**Results:**

An additional descending AVN artery was observed in 22.4% of cases and an interatrial septal branch extending to Koch’s triangle was found in 11.2% based on the macroscopic analysis—findings aligning well with the histologically verified incidence of 35.7%. These results suggest that approximately one-third of individuals exhibit an additional descending AVN artery with features resembling the normal Bonapace’s septal branch. Notably, this additional artery originates from a common trunk with the SN artery and Bachmann’s bundle branches, forming an arterial complex supplying the proximal conduction components.

**Conclusions:**

Using a large sample size, our findings highlight the anatomical and clinical importance of the arterial complex supplying the proximal conduction components, including the additional descending AVN artery. This arterial complex may include the Bonapace’s septal branch and serve as a Kugel’s collateral route in coronary occlusion. This characterisation provides basic essential anatomical data to support future research on iatrogenic conduction disturbances.

**WHAT IS ALREADY KNOWN ON THIS TOPIC**

- Transient or permanent iatrogenic arrhythmias from injury to arteries supplying the conduction system are now increasingly reported. The anatomical variations in the typical origins and courses of the sinus node and atrioventricular node (AVN) arteries have been well characterised. However, atypical AVN arteries remain poorly understood, with inconsistent terminology used to describe them. Similar branches have historically been described as Bonapace’s septal branch or Kugel’s collateral artery, which potentially supplies the AVN.

**WHAT THIS STUDY ADDS**

- This study demonstrates and visualises an additional descending AVN artery with a distinct origin, course and distribution to the compact AVN in healthy aged individuals with a reliable frequency of occurrence, based on microdissection of a large sample. This artery forms part of an arterial complex supplying the proximal conduction components.

**HOW THIS STUDY MIGHT AFFECT RESEARCH, PRACTICE OR POLICY**

- The additional AVN artery should be recognised as a potential anatomical variant within normal and collateral arterial systems. Injury to this arterial complex may contribute to transient or permanent dysfunction of the sinus node, AVN or interatrial connecting muscular bundle (Bachmann’s bundle), depending on its location.

## Introduction

 Despite over a century of investigation, the morphology of the atrioventricular (AV) conduction axis continues to be refined through integrated gross anatomical, histological and imaging techniques.^1^,^5^ However, individual variation in the cardiac conduction system (CCS) complicates the establishment of reliable anatomical landmarks for accurate localisation.^1 2^

As the CCS cannot be stained or imaged in vivo, its complex structure and precise localisation remain difficult to characterise, increasing the risk of iatrogenic conduction disorders during electrophysiological procedures. While such disorders are explicable when resulting from direct injury to the CCS itself, some occur without apparent structural damage, complicating their underlying cause. One possible mechanism involves impairment of the arterial supply to the CCS. Reported cases include sinus node (SN) artery injury during cardiac denervation or pulmonary vein isolation,^6 7^ and AV node (AVN) artery injury during slow pathway ablation for AVN re-entrant tachycardia (AVNRT).^8^,^10^ Even in the absence of complete arterial occlusion, transient complete AV block (AVB) can occur due to myocardial oedema.^9^ These findings underscore the need for a comprehensive understanding of coronary arterial anatomy supporting the CCS.

The SN artery, with up to six known anatomical variants,^11^ supplies a node located consistently within the subepicardial terminal groove—an accessible region with thin atrial musculature that facilitates arterial identification.^11^,^13^ Meanwhile, the AVN lies deeper within the tissue, making its arterial supply more difficult to identify. Due to the lack of reliable imaging markers and the macroscopic similarity of neighbouring arterial branches, AVN arteries are primarily characterised by their course towards the apex of Koch’s triangle or the central fibrous body.^14^,^16^ However, the working myocardium in this region is highly developed, unlike the SN region, and many similarly oriented branches may not actually supply the AVN.^17^ In addition, the position of the AVN within the Koch’s triangle varies significantly.^18^ Therefore, in vivo identification of the AVN artery remains challenging. Although prior reports have described the main AVN artery entering from the crux,^13 17 19^ the diversity of its other origins remains unclear.

Interatrial septal branches were often underanalysed in previous investigations of CCS vascularisation, and their anatomical and clinical relevance has remained contentious.^20 21^ Following the introduction of selective coronary angiography in the 1960s, these arteries were identified as variant interatrial septal branches in structurally normal hearts.^22^,^24^ Their consistent presence in bovine hearts has originally led to their designation as the descending septal branch of Bonapace.^25^ Subsequently, this descending interatrial septal branch has been referred to by various terms, typically describing an arterial branch mainly originating from the proximal part of the right coronary artery that courses through and supplies the interatrial septum, regardless of the conduction tissue supply or coronary occlusion (table 1).^2126^,^28^ Conversely, this artery has also been described as the pathological collateral artery of Kugel (table 1).^29^,^31^ Kugel’s artery was originally introduced as three types of variants of intercoronary anastomosis (*arteria anastomotica auricularis magna*) observed in all normal hearts.^29^ However, the term has been extended to encompass both normal and collateral branches of Kugel anastomotic arteries or network.^20 24 32 33^ Among these, Abuin and Nieponice reported that the right descending superior artery supplies the AVN in 14 out of 20 non-coronary occluded hearts (70%).^24^ Evaluating potential origins other than the right coronary artery and accurately determining the frequency of occurrence requires re-examination using a large sample size.

**Table 1:** Terminologies related to the AVN artery and their associated branches

Types	Authors	Terminology: definition/description				
		Method	Frequency of occurrence	Origin	AVN supply	Specimen remarks
**Main atrioventricular nodal artery**						
	Kawashima and Sato^17^	(Main) AVN artery: artery confirmed AVN distribution originating from coronary arteries around the crux.				
		Cadaveric dissection	101/103 (98.1%)	Both coronary around the crux	101/103 (98.1%)	No arterial occlusion
	Iwanaga *et al* ^16^	AVN branch: artery originating from coronary arteries around the crux and running through the inferior pyramidal space.				
		Cadaveric dissection	31/31 (100.0%)	Both coronary around the crux	Unconfirmed	No coronary surgical case
**Potentially AVN-distributing related branches: descending septal branch (Bonapace’s branch) and Kugel’s artery**						
	Kugel^29^	Arteria anastomotica auricularis magna: 3 types of anastomotic arteries between the proximal left, proximal right and distal right coronary arteries.				
		Cadaveric injection	Total of 50/50 (100%) for all 3 types	3 points of coronary arteries	NA	Normal hearts
	Rodriguez *et al*^26^	Descending septal branch: descending branch within interatrial septum.				
		Postmortem imaging	51/427 (16%)	Proximal right coronary or sinus	Unconfirmed	Normal and arterial occlusion
	Taylor^27^	DSA: descending branch within interatrial septum frequently supplies to AVN.				
		Postmortem imaging	50/65 (77%)	Proximal right coronary or sinus	34/50 (68%)	50% normal and 50% arterial occlusion
	Said and Mühlberger^25^	Descending septal branch (Bonapace’s branch): descending branch within interatrial septum without coronary obstruction.				
		Diagnostic imaging	A case report	Proximal right coronary	Unconfirmed	No arterial occlusion
	Abuin and Nieponice^24^	Right descending superior artery: normal descending branch originating from right coronary artery with SN artery.				
		Cadaveric injection	14/20 (70%)	Proximal right coronary	14/20 (70%)	No arterial occlusion
		Kugel’s artery (arteria anastomotica auricularis magna): abnormal descending branch originating from a large periaortic anastomosis between right and left coronary arteries.				
		Cadaveric injection	8/20 (40%)	Periaortic anastomosis	8/20 (40%)	Arterial occlusion
	Von Lüdinghausen and Ohmachi ^28^	Right superior septal artery: normal descending artery contributing to the nourishment of the interatrial septum.				
		Cadaveric dissection	27/100 (27%)	Both coronary and aortic sinus	Unconfirmed	Normal hearts
	Saremi *et al* ^20^	Right Kugel anastomotic artery: AVN branch originating from proximal right coronary artery of Kugel anastomotic arteries.				
		Cadaveric injection	Unknown	Proximal right coronary	Confirmed	Normal/abnormal
	Sanghvi *et al* ^21^	Descending septal artery (Bonapace’s branch): anomalous interatrial descending septal branch, potentially distributing to CCS.				
		Diagnostic imaging	A case report	Right aortic sinus	Unconfirmed	LAD occlusion
	Yoshikai *et al* ^33^	Kugel’s artery: anomalous interatrial descending septal branch from coronary arteries.				
		Diagnostic imaging	A case report	Proximal right coronary	Unconfirmed	Collateral branch
	The present study	Additional AVN artery: artery confirmed AVN distribution and extending from the descending branch within interatrial septum.				
		Cadaveric dissection	26/116 (22.4%)	Both coronaries	26/116 (22.4%)	No arterial occlusion

AVN, atrioventricular node; CCS, cardiac conduction system; DSA, descending septal artery; SN, sinus node.

In this study, the descending septal branch was defined as the one originating from the proximal part of the coronary artery and descending into and distributing within the interatrial septum. Additionally, when the branch extended further to the compact AVN (CN), it was classified as an additional AVN artery. Accordingly, the present study aimed to: (1) investigate an ‘additional’ arterial source to the AVN, (2) identify the peripheral distribution of a septal branch descending within the interatrial septum using a large sample of physiologically normal elderly hearts and (3) provide an anatomical foundation for future clinical electrophysiological investigations of the AVN artery.

## Methods

### Specimens

A total of 116 hearts from older individuals (mean age 85.8 years; range 57–106 years) were used. All hearts were obtained from donated cadavers injected with 7% formalin solution (2.59% formaldehyde solution) via the radial arteries. Only cadavers from donors who had provided informed consent for anatomical research at Toho University were included. Average-sized Japanese hearts, weighing 250–380 g, were selected, and markedly enlarged or hypertrophied hearts were excluded. Mild atherosclerosis, commonly observed in older individuals, was considered a physiological condition of ageing and included in the analysis. Cases with moderate or severe atherosclerosis were excluded.

### Microdissection

Microdissection of the CCS and its arterial supply to AVN was performed under a surgical microscope (OME 5000, Olympus, Tokyo, Japan). The dissection of the SN and AV conduction axis followed previously published methods.^34^ Feeding arteries to the sinus and AVN were traced from the coronary arteries and were defined as SN or AVN arteries only when their distribution could be reliably confirmed up to the respective nodes. The outer diameter of the additional artery was measured using a precision calliper (CD-15AX, Mitsutoyo, Kawasaki, Japan).

### Histology

An additional 14 intact human hearts were histologically examined. A paraffin block of the atrial septum was prepared from the central fibrous body to approximately two-thirds of Koch’s triangle. Serial sections were cut at 30–50 µm intervals and stained with Masson’s trichrome. Histological images were captured using a digital microscope imaging system (Olympus BX51 microscope with Olympus DP71 digital camera; Olympus). Images were merged using tiling software (The e-Tiling V.6.2.2.0, Mitani, Tokyo, Japan), and 3D modelling was performed using a 3D analysis software (Amira 3D V.2024.2, Thermo Fisher Scientific, Waltham, USA).

### 3D CT imaging reconstruction

To evaluate the anatomical relationship between the AV conduction axis and surrounding structures, an in situ 3D anatomical map^35^ of the AV conduction axis and its associated AVN artery was generated. Whole-body imaging was first performed using a medical CT scanner (SOMATOM Emotion 16; Siemens Healthcare, Erlangen, Germany) prior to dissection to localise the heart and CCS within the body. CT imaging parameters were set to a source voltage of 130 kV, slice thickness of 0.75 mm and reconstruction thickness of 0.5 mm. The source current was automatically adjusted to between 54 and 120 mA, depending on X-ray exposure.

For high-resolution imaging of the AV conduction axis and AV artery, additional scans were obtained using an industrial CT scanner (Naomi-CT for Industry, RF, Nagano, Japan). Imaging parameters included a source voltage of 60 kV, a current of 5 mA and a pixel size of 85 µm. Each image consisted of a 1040×1040 matrix with 16 bit greyscale values. Images were reconstructed and fused using Amira 3D software (V.2024.2, Thermo Fisher Scientific) and further processed using the 3D Slicer platform (V.5.4.0).^36^

## Results

### Identification of AVN arterial supply

**Figure 1:**
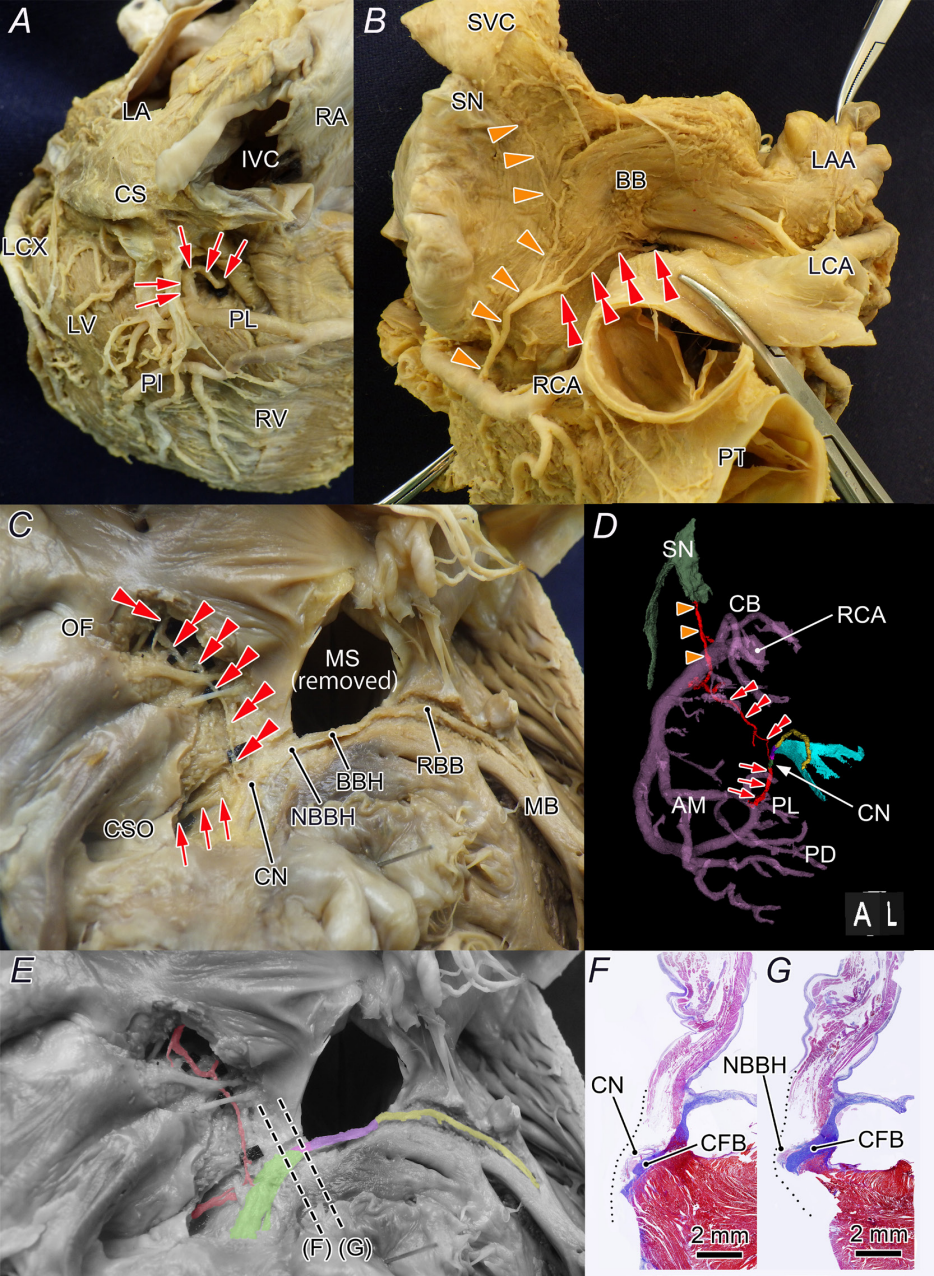
Typical origin, course and distribution of the additional AVN artery. Shown are the main AVN artery entering from the cardiac crux (**A**) and an additional descending AVN artery arising from a distinct course and sharing a common trunk with the SN artery (**B**). Within the atrial septum, the artery consistently runs near the fossa ovalis and courses inside its anterior rim towards the compact node (**C**). The SN, main AVN and additional AVN arteries are indicated by a single arrow, arrowhead and double arrowhead, respectively. The three-dimensional CT angiographic reconstruction of the right coronary artery with the SN and AV conduction axis in LAO view (**D**). Images showing the positions of the AV axes from where the slices were obtained (**E**) and their histological reconfirmation (**F, G**). AM, acute marginal branch; AV, atrioventricular; AVN, atrioventricular node; BB, Bachmann’s bundle; BBH, branching bundle of His; CB, conus branch; CFB, central fibrous body; CN, compact atrioventricular node; CS, coronary sinus; CSO, coronary sinus orifice; IVC, inferior vena cava; LA, left atrium; LAA, left atrial appendage; LAO, left anterior oblique; LCA, left coronary artery; LCX, left circumflex artery; LV, left ventricle; MB, moderator bundle; MS, membranous septum; NBBH, non-branching bundle of His; OF, oval fossa; PI, posterior interventricular artery; PL, posterolateral branch; PT, pulmonary trunk; RA, right atrium; RBB, right bundle branch; RCA, right coronary artery; RV, right ventricle; SN, sinus node; SVC, superior vena cava.

**Figure 2:**
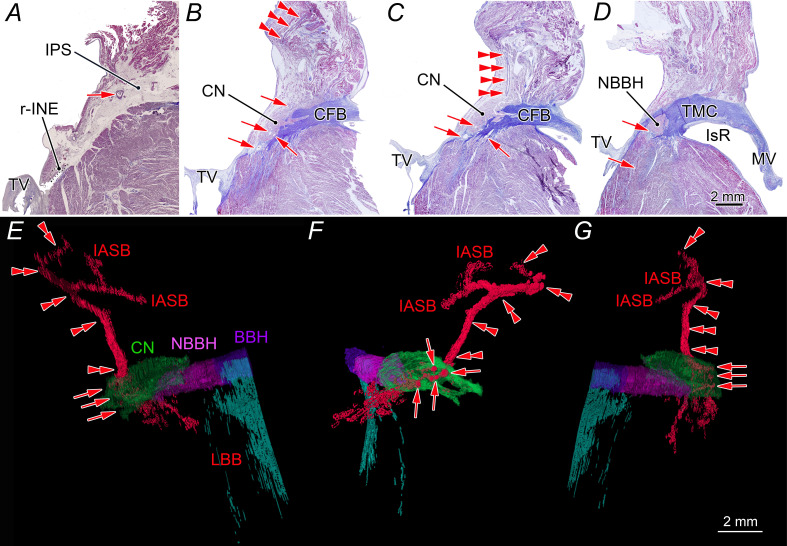
Histological evidence of dual arterial supply to the AVN (A–D), illustrated through serial sections and corresponding three-dimensional reconstructions (E–G). AVN, atrioventricular node; BBH, branching bundle of His; CFB, central fibrous body; CN, compact atrioventricular node; IASB, interatrial septal branch; IPS, inferior pyramidal space; IsR, inferoseptal recess; LBB, left bundle branch; MV, mitral valve; NBBH, non-branching bundle of His; r-INE, rightward inferior nodal extension; TMC, tricuspid-mitral continuity; TV, tricuspid valve.

To ensure accurate identification, only arteries confirmed to supply the AVN were classified as AVN arteries in this analysis of 116 structurally normal human cadavers. The main AVN artery was consistently observed among multiple similar branches entering the cardiac crux (figure 1). Additional AVN arteries with distinct origins and courses were also identified (figure 1A,B). These additional arteries originated from the proximal coronary artery as a common trunk with branches supplying the SN and interatrial connecting muscular bundle (Bachmann’s bundle (BB)) (figure 1A), descended into the interatrial septum and reached the CN in 26 of 116 cases (22.4%) (figure 1C), as confirmed via microdissection and histological analysis. Macroscopic measurements of the outer diameter of the additional AVN artery were 1.67 mm (SD ±0.28 mm; range 1.2–2.3 mm) at the common coronary origin and 0.62 mm (SD ±0.17 mm; range 0.3–0.9 mm) at the entry point to the CN. Serial histological analysis and 3D reconstruction further confirmed this additional arterial supply in five of 14 histologically examined cases (35.7%) (figure 2).

### Classification of additional descending AVN arteries

**Figure 3:**
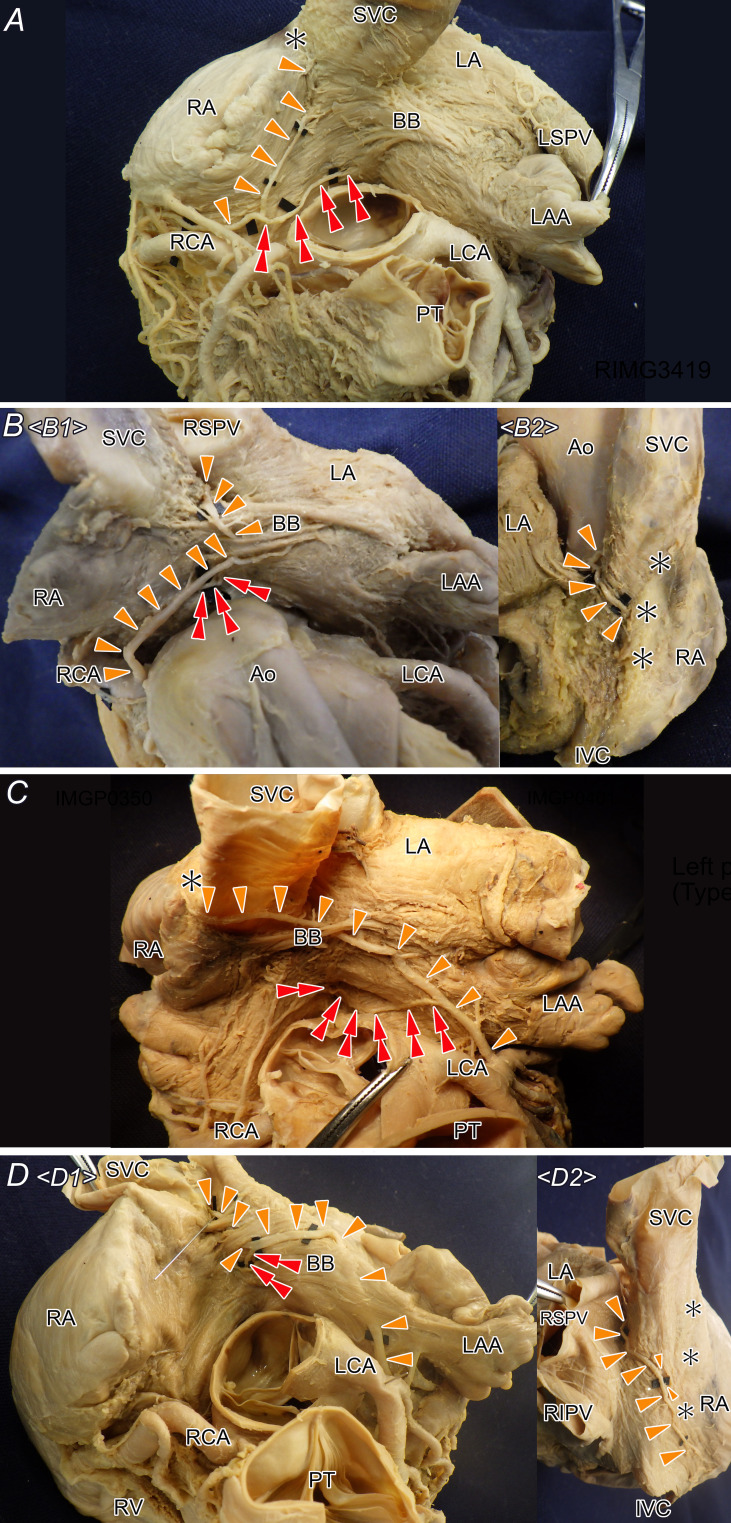
Variations in the arterial complex supplying the proximal conduction system, including the SN, BB and CN. The arterial complex was classified into four types based on the origin and course of the SN artery. The SN, main AVN and additional AVN arteries are indicated by a single arrow, arrowhead and double arrowhead, respectively. Ao, aorta; AVN, atrioventricular node; BB, Bachmann’s bundle; CN, compact atrioventricular node; IVC, inferior vena cava; LA, left atrium; LAA, left atrial appendage; LCA, left coronary artery; LSPV, left superior pulmonary vein; PT, pulmonary trunk; RA, right atrium; RCA, right coronary artery; RIPV, right inferior pulmonary vein; RSPV, right superior pulmonary vein; RV, right ventricle; SN, sinus node; SVC, superior vena cava; (A), Route 1; (B), Route 2; (C), Route 3; (D), Route 4.

In all 26 cases (100.0%), the additional AVN artery originated from the proximal parts of the coronary artery as a common arterial trunk with the SN artery and BB branches. No aortic sinus origin was observed. Their courses were classified into four types based on origin and course (figure 3):

Route 1: common trunk with the right precaval SN artery, 19/26 cases (73.1%).

Route 2: common trunk with the right retrocaval SN artery, 4/26 cases (15.4%).

Route 3: common trunk with the left precaval SN artery, 2/26 cases (7.7%).

Route 4: common trunk with the left retrocaval SN artery, 1/26 cases (3.8%).

In cases with the right coronary artery origin, the additional AVN artery originated from the craniomedial aspect of the main trunk, sharing a common trunk after branching off several conus branches. The left additional AVN artery most frequently branched from the circumflex branch and less commonly branched off from the main trunk. This branch courses for a few millimetres between the atrial muscle and the aortic sinus before entering the atrial muscle on the aortic sinus side of the atrial muscle. These branches often anastomosed with the thin branches between the left and right proximal coronary arteries. As it approached the interatrial septum, it descended, passing in the anterior part of the interatrial septum to reach the AVN. In our cases, no branches travelled the central fibrous body; all branches passed through the anterior part of the interatrial muscle, reached Koch’s triangle and then descended to the CN. In cases where AVN distribution could not be confirmed macroscopically, descending septal branches with similar origins and courses were observed in all specimens. Descending branches extending towards Koch’s triangle were identified in 13 of 116 cases (11.2%), with route 1 in eight hearts, route 2 in one heart, route 3 present in four hearts and route 4 not observed.

### Relationship with surrounding structures

**Figure 4:**
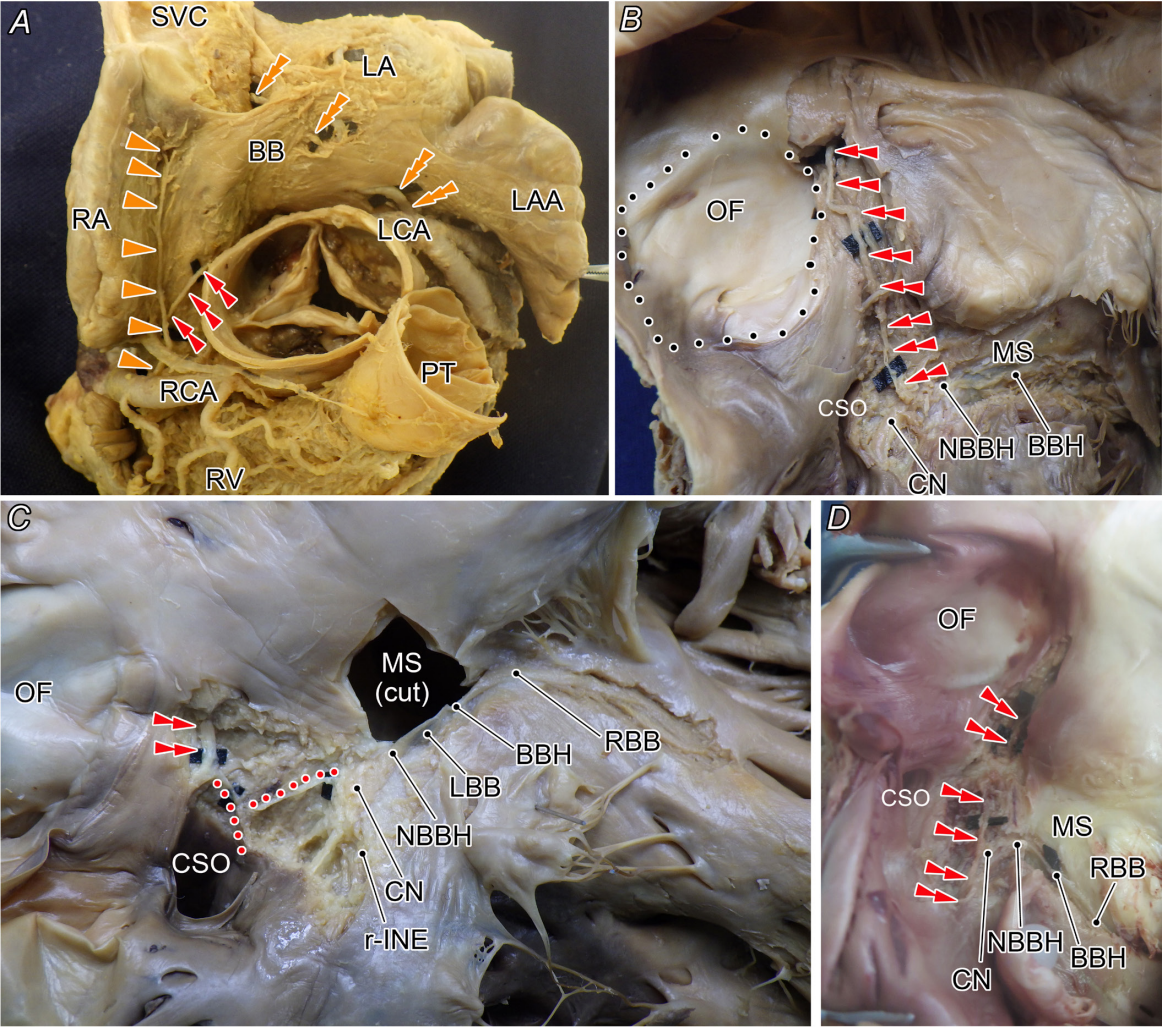
Anatomical details of the origin (**A**), course (**B**) and distribution (**C, D**) of the additional descending AVN artery. (**A**) Relationship between the origin and trunk thickness. The AVN artery may originate from either the left or right coronary artery, independent of trunk calibre. (**B**) The artery descends within the anterior rim of the oval fossa, near its anterior margin. (**C**) In most cases, the artery penetrates the CN. (**D**) In other cases, it gives rise to fine branches along the superficial endocardial surface of the CN. AVN, atrioventricular node; BB, Bachmann’s bundle; BBH, branching bundle of His; CN, compact atrioventricular node; CSO, coronary sinus orifice; LA, left atrium; LAA, left atrial appendage; LBB, left bundle branch; LCA, left coronary artery; MS, membranous septum; NBBH, non-branching bundle of His; OF, oval fossa; PT, pulmonary trunk; RA, right atrium; RBB, right bundle branch; RCA, right coronary artery; r-INE, rightward inferior nodal extension; RV, right ventricle; SVC, superior vena cava.

The arterial complex supplying proximal conduction components consistently redirected towards BB, regardless of the precaval or retrocaval origin. It then consistently branched off from the BB-associated branch. When the arterial complex originated from the right coronary artery, it tended to bifurcate into the additional descending AVN artery and a septal branch prior to bifurcation into the BB branch (figure 3A,B). Conversely, in cases with a left coronary artery origin, the complex typically penetrated the BB and then bifurcated into the additional descending AVN artery (figure 3D). While artery complexes originated from both coronary arteries, the presence of a large complex did not consistently indicate branching of an additional descending AVN artery (figure 4A).

The additional AVN artery and septal branches were relatively stable within the interatrial septum, passing within 5 mm of the anterior rim of the oval fossa in all cases (figures1C  4B).

### Peripheral distribution

In 24 of 26 cases, the additional artery penetrated the CN from its superior surface (figure 4C), whereas in two cases, fine branches passed along the superficial (endocardial) surface of the CN before bifurcating (figure 4D). Most main and additional AVN arteries penetrated or passed through the CN separately, branching into small feeding branches to the CN and eventually supplying the ventricular myocardium (figures2EG and 4D).

## Discussion

Through physical and virtual dissection, the present study clearly demonstrates the presence of an additional descending AVN artery, in addition to the main AVN artery that branches from the distal coronary artery near the cardiac crux. Notably, this is not a rare finding, as 22.4% of specimens exhibited an additional AVN artery, and 11.2% had a septal branch extending towards Koch’s triangle in macroscopic analysis, findings that aligned reasonably well with the histologically confirmed incidence of 35.7%. These results suggest that approximately one in three structurally normal hearts have an additional AVN artery with an elongated septal branch. Furthermore, the outer diameter of the additional AVN artery was 0.62±0.17 mm (range 0.3–0.9 mm) at the entry point to the CN. Although the main AVN artery was not measured in the present study, the additional AVN artery was thinner in any of the observed cases. In our previous study, the outer diameter of the main AVN artery was 0.76±0.37 mm at the entry point to the CN.^17^ However, direct comparison with the present findings is difficult due to differences in the sample used. The main AVN artery is presumed to be the primary artery based on its frequency of occurrence. The additional AVN artery supplies the AVN alongside the main AVN artery. It also supplies the SN and AVN via a common trunk with the SN artery originating from the coronary artery. As a reliable finding based on microdissection of a large sample, these results provide foundational anatomical data on the arterial supply to the CCS, which may inform for future cardiological and electrophysiological studies.

### The forgotten Bonapace’s branch and its anatomical implication

A descending septal branch—historically referred to as Bonapace’s branch—has been reported as a ‘forgotten’ artery, present in 12–85% of normal cadavers without coronary obstruction.^25^ Similarly, an AVN arterial supply via this route was reported in five of 20 (40%) cases.^24^ However, these prior studies involved small sample sizes, and imprecise identification methods may have contributed to the wide variation in incidence. Another branch with a similar course, known as Kugel’s artery, is thought to develop secondarily as a collateral pathway following occlusion of a major coronary artery.^30 31 37^ Historically, these morphologically similar branches have been inconsistently labelled as either ‘normal’ or ‘abnormal’, despite overlapping anatomical features. The characteristics of the additional AVN artery described in this study closely resemble those reported for the right superior descending artery by Abuin and Nieponice^24^ and Sanghvi *et al*.^21^ Meanwhile, unlike the right superior descending artery, this additional AVN artery can originate from either the left or right coronary artery.

Three clinical cases involving Bonapace’s branch have been reported.^38^ Two factors may explain its higher prevalence in cadaveric studies compared with clinical detection: (1) the lack of vascular tone and higher perfusion pressures during angiography, and (2) catheter positioning several millimetres distal to the right coronary artery ostium, which may obscure the origin of the branch. Previous studies suggest that Bonapace’s branch may supply the posterobasal interatrial septum and extend to the AVN and His bundle, indicating an under-recognised role in nodal perfusion.^38^

Based on our study’s large sample size, the relatively high frequency of this additional descending AVN artery supports its consideration as a normal anatomical variant, although its prominence may not be appreciable macroscopically in all cases. The criteria for its macroscopic identification did not reveal significant differences in adjacent structures.

### Differentiating the additional AVN artery from collateral pathways

These findings further demonstrate that the additional descending AVN artery follows a course similar to that of the rarely reported Kugel’s artery but differs morphologically due to its larger diameter and more tortuous trajectory.^31 33 37 39^ Whether this vessel represents a normal variant or a latent collateral pathway, it is consistently overlooked. When histological findings are considered, up to one-third of individuals have this additional artery, suggesting a dual AVN blood supply rather than a compensatory collateral vessel developing in response to ischaemia. Therefore, this additional artery may correspond to Bonapace’s branch in individuals without coronary obstruction.

**Figure 5:**
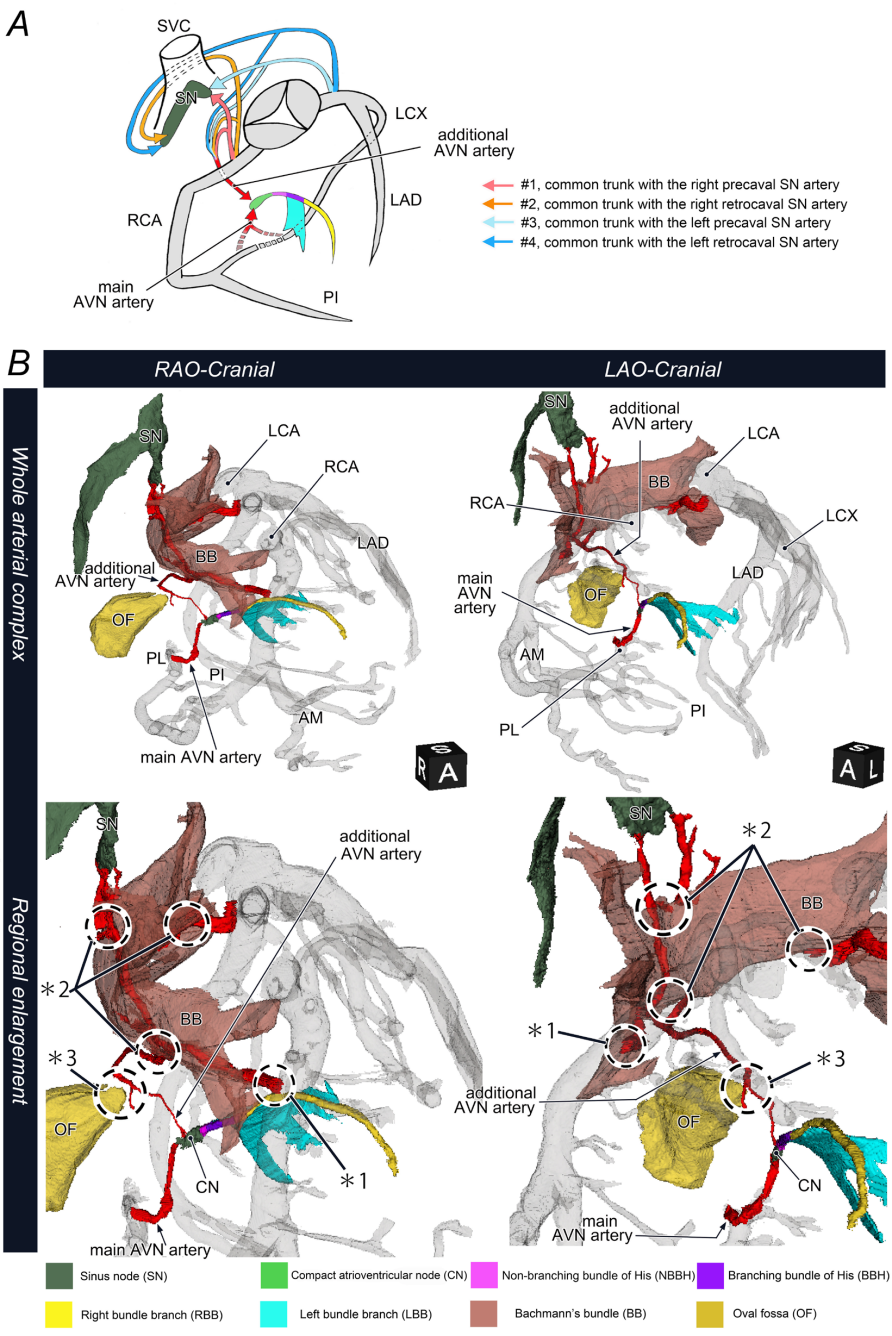
(**A**) A schematic diagram showing the origin and course of additional AVN arteries supplying CN other than the main AVN artery. (**B**) Three-dimensional CT reconstructions showing the main and additional AVN arteries, SN, AV conduction axis and surrounding structures. Left and right columns show the RAO and LAO cranial views, respectively. Lower panels show regional enlargements. The additional AVN arteries originate from the proximal coronary artery as common trunks with the SN and BB branches, most often from the RCA, and contribute to the arterial supply of proximal conduction components (*1). Their proximity to the BB branches (*2) and course through the anterior rim of the oval fossa (*3) should be noted. AM, acute marginal branch; AV, atrioventricular; AVN, atrioventricular node; LAO, left anterior oblique; LCA, left coronary artery; LCX, left circumflex artery; PI, posterior interventricular artery; PL, posterolateral branch; RAO, right anterior oblique; RCA, right coronary artery; SVC, superior vena cava.

Anatomically, this vessel is significant, as it contributes to an arterial complex that supplies the SN and BB in all specimens, and the AVN in approximately one-third (*1 in figure 5). Awareness of this artery is essential during surgical and electrophysiological procedures to prevent iatrogenic conduction disturbance due to inadvertent injury.

### Implications of the AVN arterial complex

Several studies have analysed the slow pathway ablation circuit in AVNRT using clinical and anatomical data.^40 41^ In addition, individual variations in the compact node and its inferior extension are considerable in both location and size,^18 34 42^ as is the course of the main AVN artery.^17^ Consequently, ablation lesions may inadvertently affect the main AVN artery. Furthermore, additional AVN arteries were relatively common even in individuals without coronary disease, forming part of an arterial complex. In this context, transient AVB after AVNRT ablation may be less clinically significant if a dual arterial supply is present.^9 10^

This ‘forgotten’ artery may also have implications for recent electrophysiological interventions (figure 5). For example, BB area pacing, a recent strategy for interatrial block,^43 44^ may risk occluding this arterial complex depending on the capture site and arterial course (*2 in figure 5).

In other words, only a few reports have documented AVB resulting from injury to the AVN artery during AVNRT ablation. Moreover, the significance of the additional AVN artery in BB pacing is unclear. Further data are needed to better understand its clinical complications in the future.

In transcatheter and surgical closure of secundum atrial septal defect, cardiac arrhythmias and conduction abnormalities are the most common complications: 12/442 (2.7%) in device and 9/154 (5.8%) in surgical repair.^45^ In patients undergoing closure of atrial septal defects with an Amplatzer Septal Occluder, dilation of the septum with a large expansion device has been associated with a higher risk of arrhythmia.^46^ Although right atrial remodelling is often cited as a cause, the transcatheter closure may involve a combination of factors. Conversely, in cases of new AVB following surgical closure, the underlying factors may be simpler, with injury to the additional AVN artery damage likely being the cause. The incidence of new AVB after surgical closure is six out of 135 cases (4.4%).^47^ In another report, the incidence was 23 out of 96 cases for incomplete right bundle branch block (RBBB), three out of 96 cases for complete RBBB and one out of 96 cases for complete AVB, respectively.^48^ Specifically, most conduction disturbances were transient, and permnent pacemaker implantation (PPI) was rare.^45 47^

In any case, the course of this artery near the anterior border of the oval fossa (*3 in figure 5) is noteworthy, and the relatively low incidence of AVB after AVNRT ablation and atrial septal defect (ASD) surgical repair may be related to the presence of the main AVN artery. These interpretations are based on anatomical pathways, and further clinical outcomes are necessary.

In future electrophysiological studies, some additional descending AVN artery courses may be viable targets for pacing or intervention. These anatomical findings with their implications may serve as a foundation for future clinical applications.

This study characterised the anatomy of this long-overlooked artery—historically debated as either normal or abnormal—based on a large number of cases. Photographic anatomical images and 3D reconstruction of this artery may help explain unexpected conduction disturbances and contribute to a more complete anatomical basis for electrophysiological planning.

### Limitations

As all donated cadavers were older individuals, the specimens may exhibit atherosclerotic changes, prior microinfarction or other degenerative changes such as myocardial fibrosis. Therefore, further investigation in younger adults is warranted. The image fusion methods used to construct the in situ 3D anatomical map also require further methodological and technical refinement. In future studies, the development of specialised CT techniques, such as imaging markers specific to the CCS and wide field-of-view phase-contrast CT, would be desirable.

## Conclusion

The arterial complex supplying the proximal conduction tissues is frequently present in the CCS of individuals without coronary artery occlusion and may also serve as a collateral route when occlusion occurs. Anatomical knowledge of the additional descending AVN artery is essential for anticipating and managing conduction abnormalities in both clinical and procedural settings.

## Data Availability

Data are available upon reasonable request.
